# Supplementary material for: Revealing the nervous system requirements of Alzheimer’s disease risk genes in Drosophila
Source: bioRxiv. 2025 Jul 30:2025.07.24.666383. Preprint. [Version 1] doi: 10.1101/2025.07.24.666383 (PMC12324250; doi:10.1101/2025.07.24.666383)
Supplement: Supplement 2 [file NIHPP2025.07.24.666383v1-supplement-2.pdf]

bioRxiv preprint doi: https://doi.org/10.1101/2025.07.24.666383; this version posted July 30, 2025. The copyright holder for this preprint (which was not certified by peer review) is the author/funder, who has granted bioRxiv a license to display the preprint in perpetuity. It is made available under aCC-BY 4.0 International license.

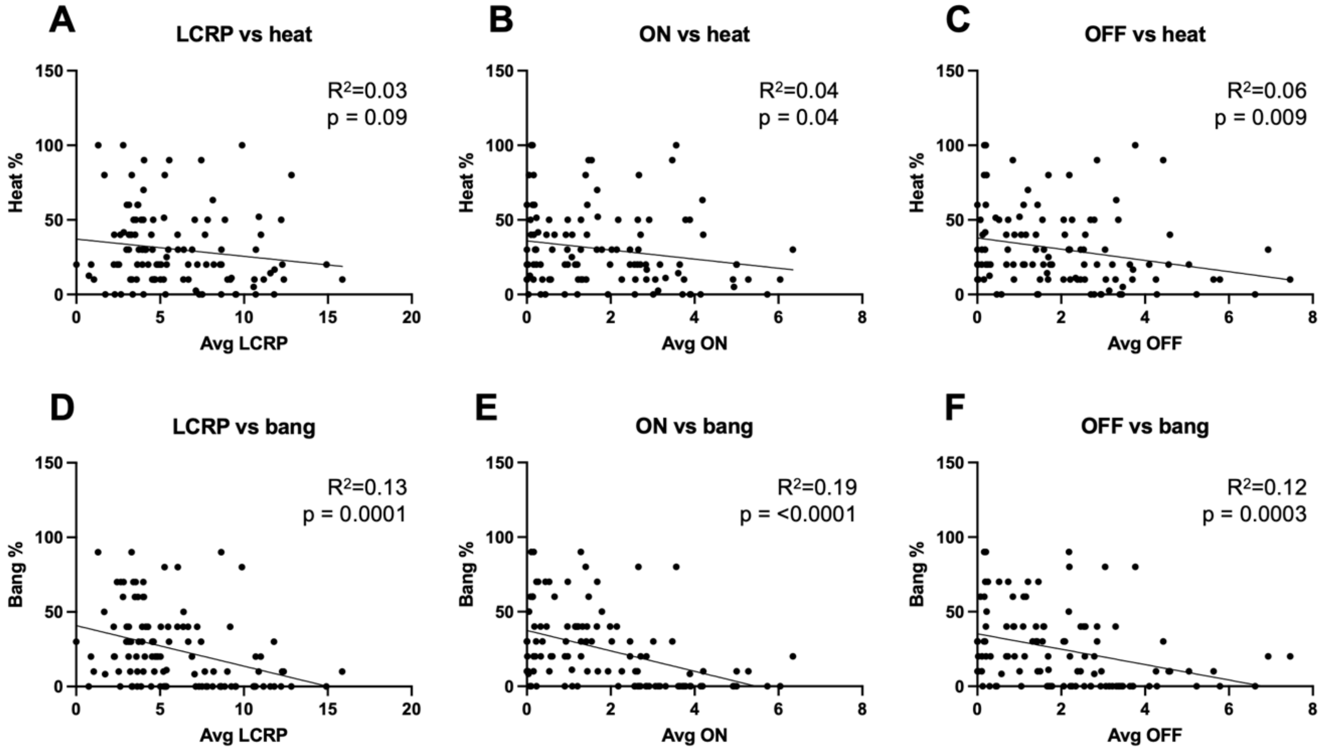

Figure S1: Negative correlation between electroretinograms and stress sensitivity. Each point represents a genotype. Average LCRP, on transient, or off transient values for each genotype are shown on the X axes. Percent impaired after heat or vortexing (bang) in the initial screen is shown on the Y axes. R^2 and P values were calculated using simple linear regression.
